# Morphometric Assessment of Confocal Laser Endomicroscopy for Pancreatic Ductal Adenocarcinoma, an Ex-Vivo Pilot Study

**DOI:** 10.3390/diagnostics10110923

**Published:** 2020-11-10

**Authors:** Bogdan Silviu Ungureanu, Daniel Pirici, Simona Olimpia Dima, Irinel Popescu, Gheorghe Hundorfean, Valeriu Surlin, Adrian Saftoiu

**Affiliations:** 1 Gastroenterology Department, University of Medicine and Pharmacy of Craiova, 200349 Craiova, Romania; adriansaftoiu@gmail.com; 2 Histology Department, University of Medicine and Pharmacy of Craiova, 200349 Craiova, Romania; danielpirici@yahoo.com; 3 Surgical Department, Fundeni Clinical Institute, 925200 Bucharest, Romania; dima.simona@gmail.com (S.O.D.); irinel.popescu220@gmail.com (I.P.); 4 Medical Clinic 1, Department of Medicine, University Hospital Erlangen, University Erlangen-Nuremberg, 91052 Erlangen, Germany; gheorghe.hundorfean@uk-erlangen.de; 5 Surgical Department, University of Medicine and Pharmacy of Craiova, 200349 Craiova, Romania; vsurlin@gmail.com

**Keywords:** pancreatic adenocarcinoma, confocal laser endomicroscopy, endoscopic ultrasound

## Abstract

Ex-vivo freshly surgical removed pancreatic ductal adenocarcinoma (PDAC) specimens were assessed using pCLE and then processed for paraffin embeding and histopathological diagnostic in an endeavour to find putative image analysis algorithms that might recognise adenocarcinoma. Methods: Twelve patients diagnosed with PDAC on endoscopic ultrasound and FNA confirmation underwent surgery. Removed samples were sprayed with acriflavine as contrast agent, underwent pCLE with an experimental probe and compared with previous recordings of normal pancreatic tissue. Subsequently, all samples were subjected to cross-sectional histopathology, including surgical resection margins for controls. pCLE records, as well as corespondant cytokeratin-targeted immunohistochemistry images were processed using the same morphological classifiers in the Image ProPlus AMS image analysis software. Specific morphometric classifiers were automatically generated on all images: Area, Hole Area (HA), Perimeter, Roundness, Integrated Optical Density (IOD), Fractal Dimension (FD), Ferret max (Fmax), Ferret mean (Fmean), Heterogeneity and Clumpiness. Results: After histopathological confirmation of adenocarcinoma areas, we have found that the same morphological classifiers could clearly differentiate between tumor and non-tumor areas on both pathology and correspondand pCLE (area, roundness, IOD, ferret and heterogeneity (*p* < 0.001), perimeter and hole area (*p* < 0.05). Conclusions: This pilot study proves that classical morphometrical classifiers can clearly differentiate adenocarcimoma on pCLE data, and the implementation in a live image-analysis algorithm might help in improving the specificity of pCLE in vivo diagnostic.

## 1. Introduction

Pancreatic ductal adenocarcinoma (PDAC) is one of the most aggressive tumors, counting as the forth most common cancer cause of death worldwide [1]. Various reasons might be incriminated for the grim prognosis including advanced stage diagnosis, resistance to conventional oncologic treatment, early vascular and neural invasion as well as new studied genetic alterations or tumor microenvironment [2]. The growen burden of this disease still falls to surgery, even though there is only a 20 to 25% survival rate in a resectable stage. Therefore, an accurate staging process that may assure a precise resection margin may be related to a better prognosis.

Confocal laser endomicroscopy (CLE) was introduced as a ground-breaking technique for gastrointestinal lesions by providing in vivo, real-time subsurface pathologic diagnosis [3]. Based on a laser-beam excitation, tissue-sequences are generated due to tissue reflectance or fluorescence. Moreover, the use of a dye enhances the clinical utility and reproduces similar pathological images [4,5]. Probe based confocal laser endomicroscopy (pCLE), with various miniprobes is available as a prehistopathological asset for different types of lesions offering real time images for the physician.

There are only few clinical trials as reference for CLE imaging in pancreatic masses, especially on cystic lesions [6,7]. Regarding solid pancreatic masses, CLE was introduced in the aid of fine needle aspiration (FNA) with the purpose to improve the accuracy rating of differentiating between benign and malignant tumors [8,9,10,11]. The so far published studies tried to emphasize imaging characteristics of pancreatic malignant lesions. However, interobsever variability, risk stratifications as well as the lack of standard reference when performing CLE should be considered as critical factors for diagnosis. Thus, a better correlation with histology might be useful to successfully point out specific CLE atributes.

These flaws may be surpassed when comparing CLE acquired images with cross sectional pathology on ex-vivo surgical freshly removed pancreatic masses. The aim of this pilot study was to provide a better understanding of PDAC endomicroscopy characteristics and to compare them in a direct manner with histopathology in an endeavour to find putative image analysis algorithms that may lay the grounds for future clinical studies.

## 2. Materials and Methods

### 2.1. Patients Selection

Twelve patients diagnosed with PDAC based on contrast enhanced endoscopic ultrasound (CEUS) and fine needle aspiration (FNA) cytology analysis were enrolled in this pilot study. Patients were prospectively selected and after EUS tumor staging, surgical criteria was established for resectable and borderline resectable tumors according to the National Conprehensive Cancer Network Guidelines (NCCN) and European Society of Medical Oncology (ESMO). After tumor removal, specimen samples were harvested from tumor as well as from normal pancreatic tissue as controls from the resection margins, followed by CLE analysis and histopathologic comparison. All patients signed an informed consent prior to study inclusion and the layout was approved by the local Ethics Committee of the University of Medicine and Pharmacy of Craiova (No 33 from 20.03.2018).

### 2.2. Ex-Vivo Assessment

All images were acquired with a ColoFlex UHD–Cellvizio Confocal Miniprobe (Mauna Kea Technologies, Paris, France), with a 488 nm wavelength. This probe produces a field view of 240 μm and has a confocal depth ranging from 55 to 65 μm. We chose this probe because it is thicker than the desirable AQ-Flex 19 which is used for pancreatic lesion’s assessment, thus handling was easier in an ex-vivo setting.

**Figure 1:**
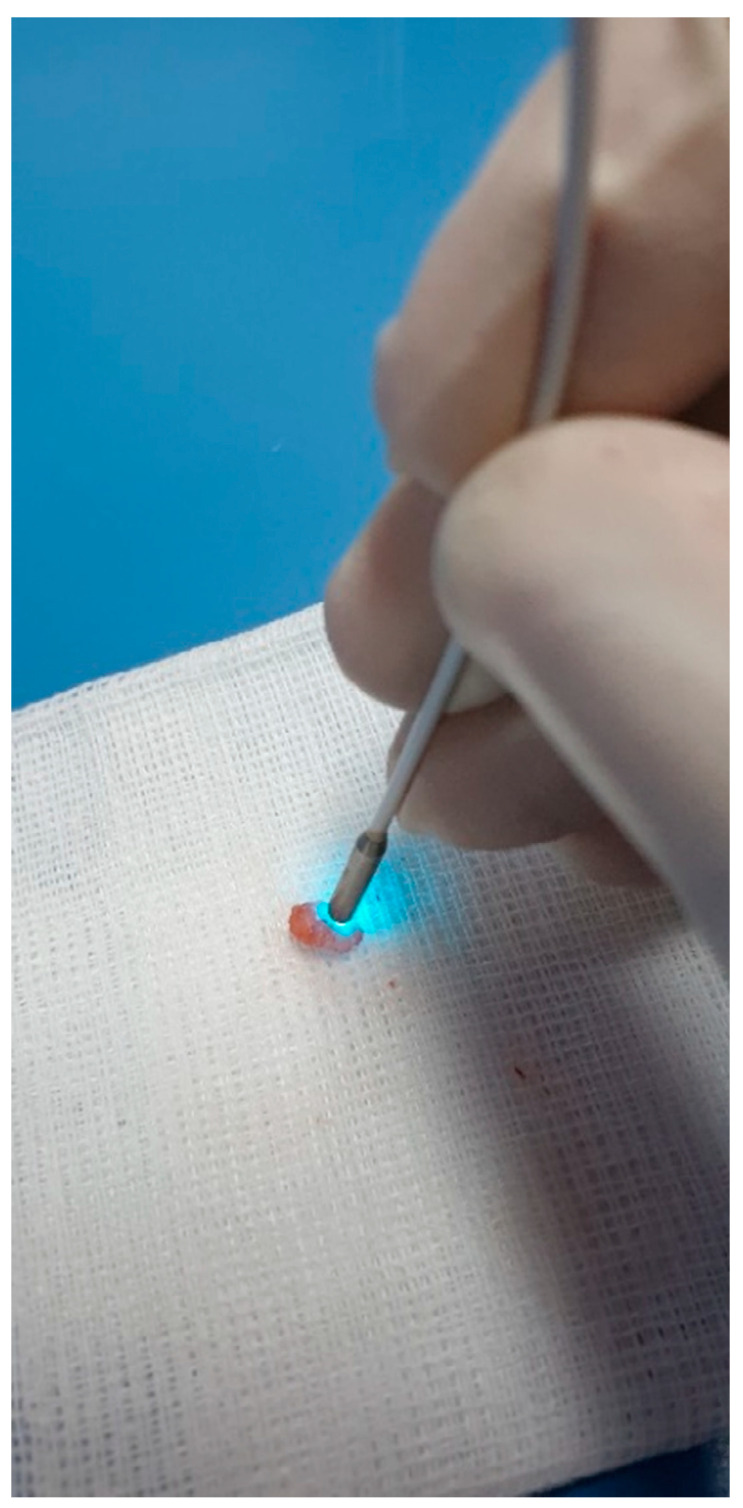
Ex-vivo tissue CLE imaging acquired after acriflavine exposure.

To simulate a human use application as closely as possible, the dedicated samples were washed with serum and incubated for 1 min in acriflavine hydrochloride 0.025%. After a 5 min drying process, the harvested tissue was set in place in a dedicated design. The confocal miniprobe was used in direct contact with the specimen’s surface with gentle moves covering the entire area (Figure 1). Dynamic real-time images were immediately observed and recorded within the CLE system.

### 2.3. Histopathological Criteria

Specimens were sent for pathologic confirmation. For a better comparison between normal and pathologic tissue we used Pan Cytokeratin AE1/AE3, which is a polyclonal antibody that allows qualitative assessment and may be extended to a morphological study. The immunohistopathological study also included CEA and Ki67 assessment. Tissue fragments were incubated for 1 h at 37 °C, fixed and sent for paraffin embedment. We acquired 40× images from both normal and all the available casuistry, with an average of 5 images/slide, with the same illumination and exposure settings on a Nikon 90i microscope.

### 2.4. Image Analysis

Video recordings from the CLE system allowed to stop and select the desired frames. 5 CLE images were selected by the same physician who performed the procedures from every patient with tumor and normal tissue and compared with the pathologic corespondant. A dedicated software (Image Pro-Plus AMS, Media Cybernetics, Bethesda, MD, USA) for image analysis was used to compare both settings. After uploading the images a grey/color profile and a region of interest (ROI) was considered, followed by the analysis of several morphometric parameters from each image: (1) total area of the signal, (2) hole area and (3) heterogeneity—as indicators of inner irregularity, (4) Integrated optical density (IOD), as a measure of pixel intensity and area, (5) roundness and (6) fractal dimension as measures of edge and silhouette-related irregularity, (7) ferret as a measure of average and maximum diameters of the ROIs and (8) clumpiness, as an indicator of pixel variations in the inner areas of the ROIs.

### 2.5. Statistic Analysis

The generated data was run by Microsoft Office Excel^®^ (Microsoft, Redmond, Washington, DC, USA). The results from every parameter were defined as averages and means and t-test was performed with statistical significance achieved at a *p* value of <0.05.

## 3. Results

This section may be divided by subheadings. It should provide a concise and precise description of the experimental results, their interpretation as well as the experimental conclusions that can be drawn.

Over the study period, twelve patients were prospectively enrolled after being diagnosed with resectable or borderline resectable PDAC including 8 males and 4 females with a median age of 65.4 years old (53–78). The average tumor size was 30 mm (20–70 mm) with the majority of tumors being located within the head of the pancreas (58.3%) followed by the tail of the pancreas (33.3%) and body (8.3%). Vascular resection was performed in 7 cases and 4 patients had previous oncological treatment. Two small resection specimens were harvested from each patient, from the plain tumor site as well as safety resection margins (Table 1).

**Figure 2:**
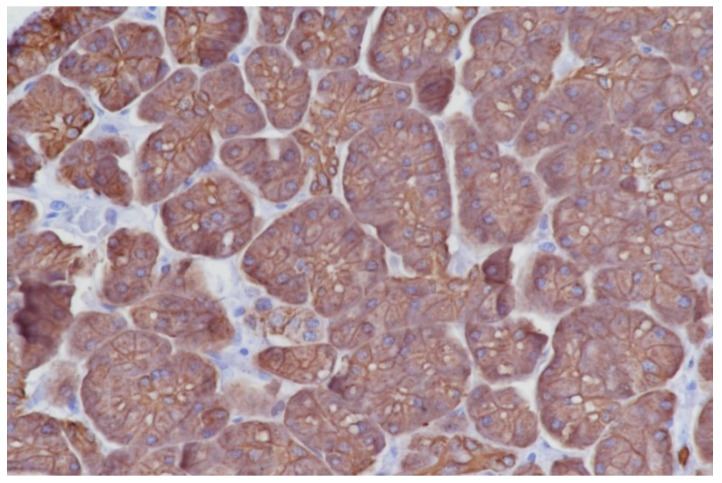
Normal pancreatic tissue on histopathology analysis, showing normal pancreas acini. Immunohistochemistry for Pan cytokeratin AE1/AE3, 40×.

**Figure 3:**
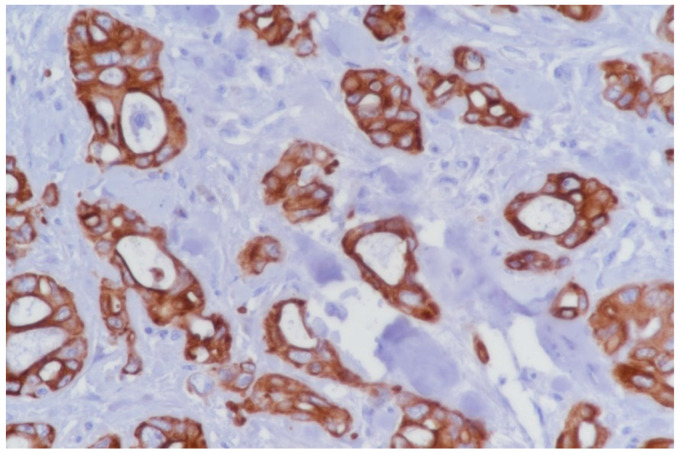
Histopathological images showing infiltrating proliferations in ductal carcinoma of the pancreas. Immunohistochemistry for Pan cytokeratin AE1/AE3, 40×.

**Figure 4:**
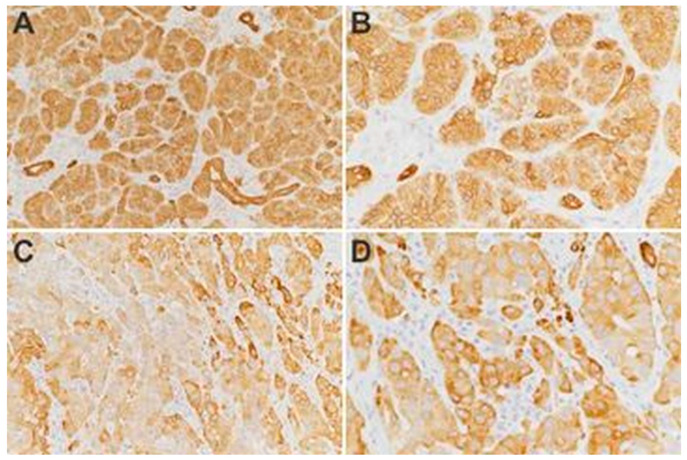
AE1/AE3 cytokeratin staining on control pancreas tissue (**A**,**B**) and ductal adenocarcinoma cases (**C**,**D**); **A**,**C**—20×, **B**,**D**—40×.

To portrait an efficient denominator that could differentiate between normal pancreatic tissue and PDAC we firstly compared the parameters on histopathological images. Overall morphology changes were assessed after AE1/AE3 pan-cytokeratin staining enhancing different structures from the regulated round acini and ducts to the tortous and irregular shapes in PDAC cases (Figure 2, Figure 3 and Figure 4). All the denominators’ values were first averaged per slide, then per patient, and in the end control cases were compared with tumor patients utilizing a simple Student t test. Total area and hole area did not show significant differences (*p* = 0.0749 and respectively 0.0820), while IOD, roundness and heterogeneity showed significant differences (*p* < 0.05), and the perimeter, fractal dimension, ferret and clumpiness showed extremely significant differences (*p* < 0.001) (Table 2).

**Figure 5:**
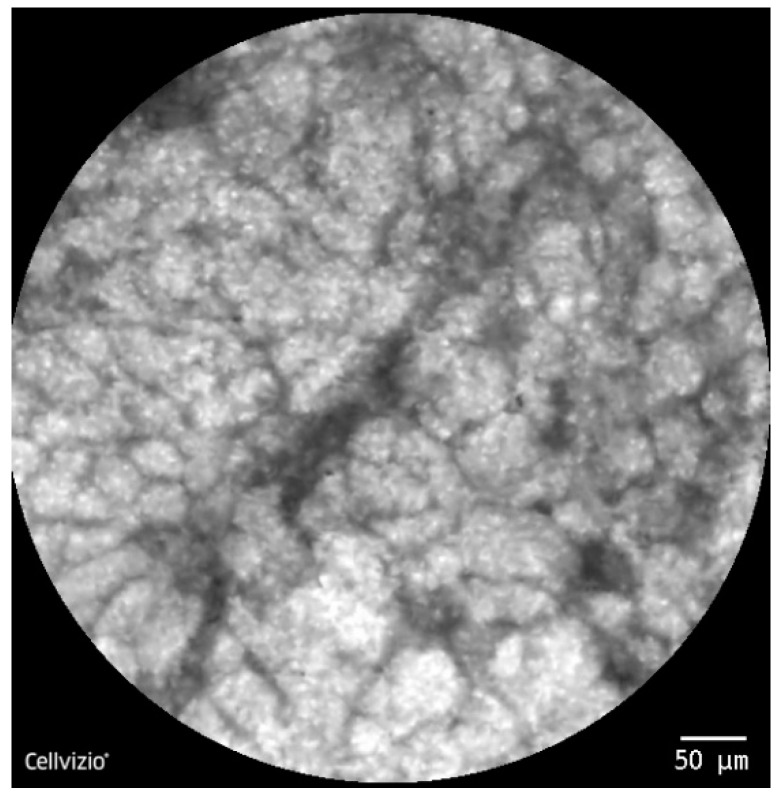
CLE image with lobular structures, clearly demarcated, showing a regular pattern representing the pancreatic acini.

**Figure 6:**
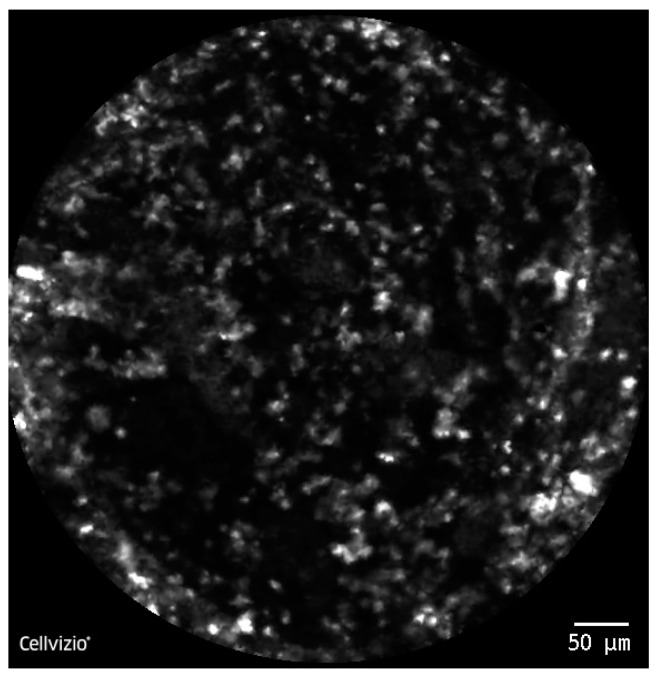
CLE image—disorganized structures with different sizes nuclei and the presence of dark aggregates representing PDAC.

For the pCLE imaging, we firstly defined the characteristics of normal tissue in comparison with PDAC samples. Benign lesions were presented as lobular structures, clearly demarcated, showing a regular pattern representing the pancreatic acini and conjunctive tissue randomly disposed (Figure 5). Malignant lesions were characterized by disorganized structures with different sizes nuclei and the presence of dark aggregates (Figure 6). pCLE parameter analysis followed the same pattern when comparing endomicroscopy normal tissue and endomicroscopy tumor tissue. Clumpiness did not show any correlation (*p* = 0.35), fractal dimension did not show significant differences (*p* = 0.07), whereas hole area showed significant correlation (*p* < 0.05) and area, IOD, perimeter, roundness, Ferret max, Ferret min, Heterogeneity revealed high differences (*p* < 0.01).

## 4. Discussion

PDAC represents one of the major therapeutic challenges due to the late diagnosis or resistance to chemotherapy. Early diagnosis ensures surgical removal and prolongs patient’s survival.

CLE has evolved as a high potential imaging tool for real-time diagnosis and staging of different gastrointestinal lesions [12]. By providing optical biopsies, with additional information including blood flow and contrast up-take or leakage, ensuring resection margins in different cancers or inflammatory bowel disease therapy assessment, the use of CLE is on an ongoing trend, fostering a strong incentive for research. Alongside with many studies, CLE accuracy has proven to increase to over 90% if standardized parameters are developed for lesions assessment [13,14,15,16]. So far, in pancreatic tumors, CLE has been validated as a valuable tool for diagnosis and risk stratification of pancreatic cysts with the ability of differentiating between cystic lesions [17]. However, solid pancreatic tumors CLE diagnosis can be particularly challenging since there is limited data, with no standardization of practice or lesions characteristics.

Our pilot study focused on using morphologic parameters assessment of PDAC ex-vivo CLE images, without direct characterizing the lesions, which may correlate to a more accurate real-time diagnosis. Tangential CLE images were backed up by standard histopathology, and assembled in a larger picture by highlighting different cellular characteristics using a specific designated software.

We included 12 consecutive patients diagnosed on EUS-FNA with PDAC, which also met the surgical criteria for resection. While there are some studies that use nCLE for solid pancreatic masses, our pilot study focused on comparing freshly resected specimens with control samples from the same patient. While others have used fluoresceine as a contrast agent, we decided to use acriflavine hydrochloride, which has the ability of nuclei enhancement and allowed us to describe the distinctive features of cellular appearance between normal cells and adenocarcinoma. Also, quantifying the mucleo-cytoplasmatic rate is one of the hallmarks used for cytopathologic diagnosis of PDAC. Acriflavine has been used in clinical practice as CLE dye, whilst the advserse events are still debatable [18,19,20,21,22].

The morphologic features from both normal and tumor tissue were directly compared by using a specific software, thus excluding the human factor which may lead to different judgement of encountered lesions. We successfully compared the tissue and proved that pCLE images were closely tied to the pathologic assessment. The dominators used were correlated within the compared sections proving that an assembly of this type might ensure a potential diagnosis directly in vivo in PDAC. This might be a rather attractive way to directly visualize the invaded tissue, thus ensuring potential resection margins and moreover be a more reliable method to check for restant malignant cells after tumor removal.

While this is only a pilot study to emphasize similar patterns between pCLE and histopathology images in PDAC, the use of a contrast agent is of great importance. We decided to use an ex-vivo setting and acriflavine as a contrast agent to better point out cellular features and also ensure enough time for each examination. Two other studies [9,11] focusing on nCLE and solid pancreatic tumors showed similar results on describing the tissue characteristics while using fluorescein as a contrast agent. The first study focused on directly comparing EUS-nCLE results with EUS-FNA interpretation with images being directly analyzed by physicians [9], whereas the second study was based on a prospective blind situation where the results were reviewed without any knowledge of the diagnosis [11]. Results were rather similar, especially when interpreting malignant tissue. However, in none of these situations there was a direct comparison with histopathology results.

This proof-of principle study showed that simple algorithms can differentiate large histological structures, without considering sub-cellular details. It was conceivable that ex-vivo or even in-vivo staining with fluorescently labeled contrast agent might yield detectable differences on images captured with pCLE, where large ducts and acini should be visible even on lower resolution images.

We suggest this pilot study as a launching pad for future research of rapid on-site evaluation of pancreatic tumors and also for building a link for new options to be used in daily practice. Several limitations inherent in our research should be noted such as the small number of patients, the fact that we only focused on PDAC and most of all that we did not provide an in vivo diagnosis by using acriflavine as contrast agent, our study should be interpreted more as a fundamental study to lay the basis for future development in CLE. Thus, it is clear that most of these morphological denominators can be used to create specific and different patterns for both the normal histology and overall ductal adenocarcinomas (which were not considered separately based on grading or disease extension). An automated image analysis algorithm, or a trainable neural network using these parameters would thus be able to appreciate the probability of viewing a tumor or a control tissue without the intervention of the user, once the images have been acquired.

CLE is a valuable tool, which offers new opportunities for early and live diagnosis of pancreatic tumors. This preliminary study provides another point of view for PDAC diagnosis by analyzing morphometric parameters, and may play a significant role in faster and more reliable analysis of in-vivo tumoral tissue.

## 5. Conclusions

CLE is a valuable tool, which offers new opportunities for early and live diagnosis of pancreatic tumors. This preliminary study provides another point of view for PDAC diagnosis by analyzing morphometric parameters, and may play a significant role in faster and more reliable analysis of in-vivo tumoral tissue. Furthermore, clinical usage of this exiting technology could be expanded as tiny needle-based probes (nCLE) can be utilized during 19G EUS-guided fine needle aspiration (EUS-FNA) procedures.

## Figures and Tables

**Table 1:** Patient’s characteristics that followed surgery.

Patient’s characteristics	
Patients, n	12
Male, n	8
Mean age, years old (min–max)	65.4 (53–78)
FNA with positive citology for PDAC	12
Tumor location	
Head of the pancreas	7
Body of the pancreas	1
Tail of the pancreas	4
Tumor size (min–max)	30 mm (20–70 mm)
Surgical procedure	
Whipple	8
Distal pancreatectomy	4
Vascular resection	
performed	7
Not performed	5
Previous chemotherapy	4

**Table 2:** Morphologic parameters assessment.

	Histology Normal Tissue vs. Histology Tumor	Endomicroscopy Normal Tissue vs. Endomicroscopy Tumor	Histology Normal Tissue vs. Endomicroscopy Normal Tissue	Histology Tumor vs. Endomicroscopy Tumor
**Area (*p*)**	0.07493	0.00000	0.00001	0.02418
**Hole Area (*p*)**	0.08205	0.00491	0.04719	0.00000
**IOD (*p*)**	0.00046	0.00000	0.00001	0.39271
**Perimeter (*p*)**	0.00000	0.00000	0.00000	0.00024
**Roundness (*p*)**	0.01200	0.00039	0.00005	0.00000
**Fractal Dimension (*p*)**	0.00000	0.07137	0.16772	0.10198
**Ferret max (*p*)**	0.00000	0.00000	0.00000	0.00001
**Ferret mean (*p*)**	0.00000	0.00004	0.00000	0.00001
**Heterogeneity (*p*)**	0.00596	0.00004	0.00003	0.00002
**Clumpiness (*p*)**	0.00009	0.35528	0.00367	0.00133
